# Study on Some Thermal and Electrical Properties of Basalt Fabric Modified with Metal and Ceramics as a Result of Magnetron Sputtering

**DOI:** 10.3390/polym11122087

**Published:** 2019-12-13

**Authors:** Pamela Miśkiewicz, Iwona Frydrych, Magdalena Tokarska, Wojciech Pawlak

**Affiliations:** 1 Institute of Architecture of Textiles, Faculty of Material Technologies and Textile Design, Lodz University of Technology, 90-924 Lodz, Poland; pamela_miskiewicz@wp.pl (P.M.); iwona.frydrych@p.lodz.pl (I.F.); 2 Institute of Materials Science and Engineering, Faculty of Mechanical Engineering, Lodz University of Technology, 90-924 Lodz, Poland; wojciech.pawlak@p.lodz.pl

**Keywords:** basalt fabric, magnetron sputtering, surface modification, thermal properties, electrical properties, CIELAB system

## Abstract

The main aim of the research was to compare the values of some thermal and electrical parameters obtained for a basalt fabric modified with the metal and ceramics coatings. The surface modification of basalt fabric was made by using a magnetron sputtering technique. Chrome and zirconium(IV) oxide coatings were deposited on the fabric surface. The thermal and electrical properties of selected fabrics were determined. In order to assess the comfort properties of fabrics, the thermal resistance of materials was analyzed. Instrumental color measurement was used for an assessment of the surface of modified and unmodified basalt fabric. Using a non-contact digital color imaging system, DigiEye, an original method of samples surface analysis was presented. As a result of research, the modification of basalt fabric surface for applications in a hot work environment enabled the improvement of thermal properties in relation to the references samples. The first level of protection against contact heat for a contact temperature of 100 °C was obtained for the zirconium(IV) oxide-modified basalt fabric. The first level of protection against radiant heat was obtained for all samples. The highest value for the heat radiant resistance was obtained for the chrome-modified basalt fabric.

## 1. Introduction

Nowadays, the textile sector strives to develop innovative technologies or to combine different techniques and materials to obtain a new material of different or better properties. Modern solutions appearing in the textile industry are mainly intended to provide the user with the comfort and functionality of the product under specific environmental conditions. To prevent various types of hazards in a hot microclimate environment, specialist clothes are required, particularly protective gloves [1,2,3]. The protective gloves should meet the basic standard [4]. This standard does not directly apply to the protective properties of gloves, and therefore should not be used as a separate standard, but only in conjunction with the relevant European standards. In the case of protective gloves, in particular gloves protecting against hot factors, it is difficult to reach a compromise between providing adequate protective and functional properties. Usually, a higher level of protection in terms of thermal resistance involves the use of several layers of different materials. These layers significantly reduce the efficiency and ability to manipulate the fingers. Therefore, it is advisable to replace several layers with one textile material with a coating that meets the requirements for protective gloves. Therefore, the basalt fabric was selected for testing. Basalt fibers and products made of them are characterized by a low thermal conductivity, low moisture absorption, and good thermal resistance [5,6,7,8]. These fibers have high thermal resistance, which enables them can be used in the temperature range from −260 to 800 °C. Due to the non-flammable properties that characterize basalt fibers, the materials made of them are resistant to flames for a very long period of time. In addition, they are resistant to corrosion, UV radiation, and the action of microorganisms. Products made of basalt fibers do not emit toxic products in reaction with the air or water. Basalt fabric is characterized by good thermal and mechanical properties. However, basalt fabrics used without any coating may cause irritation to the skin, respiratory track, and eyes [9,10,11].

The BAGLO project led by the Lodz University of Technology concerned developing a textile package for protective gloves, the task of which was the protection against hot factors while providing protection against mechanical factors. In order to develop a new type of gloves, the protective fabrics made of basalt fibers were used. These included uncoated basalt fabrics and basalt fabrics, to which aluminum foil has been glued using a special adhesive mean. None of the selected basalt fabrics intended for gloves protecting against the effects of hot factors showed resistance to contact heat for the contact temperature of 250 °C. In the case of heat-resistance tests, the highest (fourth) efficiency level was obtained for all samples of aluminized basalt fabrics. One of the disadvantages of the produced aluminized basalt fabric was the abrasion and cracking of applied coating [1,12], which led to a loss of protective properties and durability of gloves. Due to the above-described negative aspects of the conducted research, this work concerns the modification of basalt fabric in a different way (without) gluing the aluminum foil, but by putting chrome or zirconium (IV) oxide directly on the fabric surface.

In order to modify the basalt fabric surface, one of the PVD (physical vapor deposition) processes was applied: magnetron sputtering. It relies on the creation of atoms, atom clusters, or ions in a gas phase by working gas (Argon) ionization in a high voltage potential and them striking the surface of the target. In particular, magnetron sputtering is one of the glow discharge types, with a crossed electric and magnetic field, which allows for electron trapping in a sputtering zone. This is responsible for the higher flying time of electrons and results from the higher amount of sputtering ions in a near-to-target zone. This, in turn, causes the high sputtering rate of the target and faster deposition in comparison to diode sputtering. Magnetron sputtering can be done with the use of metallic targets (i.e., chromium or zirconium) in a case of pure metallic coatings. In order to obtain ceramic phases (nitrides, oxides, or carbides) to the working atmosphere reactive gas (nitrogen, oxygen, or carbon-containing gas, such as for example methane or acetylene) should be introduced. Such a process is called reactive magnetron sputtering (RMS) and is commonly used for the deposition of coatings that improve hardness, corrosion resistance, tribological, or decorative properties as well as other physical properties of products used in various fields of industry. The versatility of magnetron sputtering allows for the constitution of a coating onto a very broad range of shapes and sizes [13].

Magnetron sputtering is a technique that allows the improvement of fabric thermal properties. A two-layer coating—aluminum (Al) with silicon dioxide (SiO_2_)—and a three-layer coating—silicon dioxide (SiO_2_)/aluminum (Al)/silicon dioxide (SiO_2_)—were deposited on the aramid fabric surface [14]. In this way, the radiant resistance of fabric was improved. The thermal and physical properties of nylon fabric subjected to metal sputtering—i.e., aluminum, copper, and nickel—were investigated [15]. It was found that the sputtering treatment could give significant effects to the heat transfer of nylon without much loss in its shear and bending property, as well as water vapor transmission. The sputtered aluminum fabric was also studied for changes in its heat transfer [16]. Aluminum was applied onto four textile substrates: nylon, polyester, cotton blend with 50/50 polyester, and shape memory polyurethane. Basic fabrics and fabrics modified with aluminum coating indicated the different thickness and thermal conductivity. The cotton–polyester blend (50/50) showed the lowest heat transfer coefficient. 

Conductive paths, antennas, light-emitting diodes, and detector systems are used on the surface of textile materials, mainly used for recording physical and chemical changes in nature. There are many scientific publications on the modification of nonwoven, woven, and knitted fabrics as a result of a physical vapor deposition process intended for the use in textronics. One of the most interesting publications is the study of the resistance of metal coatings used in textronic systems for mechanical deformations [17]. The work uses a composite Cordura substrate consisting of nylon fibers, which are coated with polyurethane foil. The 99.9% pure silver was deposited on the selected substrate. As a result of the study, it was observed that thin electrically conductive coatings obtained by the process of physical vacuum deposition without protecting their surface against destructive factors showed very good resistance to cyclical mechanical deformations. Besides, the authors stated that such structures can create flexible components that can be used in many areas of textronics and electronics.

The authors are first of all interested in improving the resistance to contact heat at a contact temperature of 250 °C, in order to be able to use the fabric in gloves protecting against the flame and hot factors. Gloves should protect against high temperatures outside, while maintaining a comfortable temperature inside. It is difficult to reach a compromise between providing adequate protective and functional properties.

To improve thermal properties, two different coatings (ceramics and metal) were chosen for the basalt fabric modification, i.e., the zirconium(IV) oxide and chrome coatings. Unmodified and modified basalt fabrics were tested for their thermal and electrical properties to be able to use the fabrics in gloves protecting against the flame and hot factors and damage by an electrostatic discharge. The research was aimed at increasing the contact heat resistance without reducing the radiation heat resistance. The authors also wanted to compare the values of some thermal and electrical parameters obtained for basalt fabric modified with metal and ceramic coatings.

The khaki color of basalt fabric has been changed due to the coating deposition. Instrumental color measurement was used as the method providing an objective data with a sufficient accuracy and repeatability for the color assessment of basalt fabric and basalt fabric modified with the chosen coatings. It is useful in the case of measurements of surface textures [18], especially the textile material surface [19,20,21]. Using the non-contact digital color imaging system DigiEye, an original method of samples surface analysis was elaborated and presented.

## 2. Materials and Methods 

### 2.1. Samples

A fabric made of basalt yarns was selected for tests. It was subjected to the deposition of coatings process by the magnetron sputtering with the use of chrome or zirconium(IV) oxide. In the case of chrome coating, an increase in the radiation heat resistance is expected due to its shiny surface. The coating thickness should be chosen, so that the sample is still in the group of anti-static materials, which is important for gloves (such as for example those intended for foundry workers). In the case of zirconium(IV) oxide coating, an increase in the contact heat resistance is expected due to its matte surface, dielectric properties, and very small thermal diffusion coefficients.

Selected coatings were deposited on the twill 2/2 weave fabric of the following structural parameters: mass per square meter 398 g/m^2^, thickness 0.55 mm, apparent density 724 kg/m^3^, and 8 × 9 threads per 1 cm^2^. Using an Olympus SZX10 stereo microscope (Tokyo, Japan) and Olympus Stream Start software (ver. 1.5, Tokyo, Japan), images of basalt fabric and coated basalt fabrics with a chrome coating thickness of 20 µm and zirconium(IV) oxide coating thickness of 18 µm were captured and presented in Table 1.

### 2.2. Deposition of Coatings on the Basalt Fabric Surface

The magnetron sputtering deposition of pure chromium or zirconia coatings was performed with the use of industrial vacuum unit URM 079 (Minsk, Belarus) equipped with one magnetron plasma source [9]. Usually, solid samples are cleaned in the solution of detergent and next in organic solvent (acetone or isopropyl alcohol) with use of ultrasound cleaner. In this case, because of the internal structure of the fabric and capillary collection of liquids inside the fiber, this step was avoided. The basalt fabric was cut to the dimension needed for heat transfer and other measurements and mounted to the sample holder with special care, in order to prevent any fat contamination on the surface. Besides the fabrics samples, there were also mounted monocrystalline silicon samples for the determination of thickness and chemical composition.

The deposition process has started with pumping of the vacuum chamber to the pressure of 2 × 10^−3^–3 × 10^−3^ Pa. After reaching the base pressure, the 5 N argon pressure was set to about 0.5 Pa. One axis rotation has started, and 4″ magnetron has been launched with the power and time, as shown in Table 2. For the chrome coating, a pure chromium target with 99.99% purity was used. In the case of zirconium oxide, there was a conducting reactive process with the use of a pure zirconium (99.5%) target and the addition of 18 sccm of oxygen (5 N) flow. The partial pressure of oxygen was about 4 × 10^−2^ Pa.

### 2.3. Chemical Composition and Thickness Determination

Scanning electron microscopy (SEM) and X-ray energy dispersion spectroscopy (EDX) were used to study chemical composition (in this case, the distribution of elements in the sample) for an assessment of chrome and zirconia coatings deposited on the surface of basalt fabric. For research, the JEOL scanning electron microscope model JSM-6610 LV (Jeol, Japan) with special software EDS AZtecEnergy (ver. 3.1, Oxford Instruments, High Wycombe, UK) was used. The thickness of coatings was measured with the use of SEM pictures of the fracture of the silicon witness sample. The accuracy of the thickness determination was apprised as ±100 nm.

### 2.4. Protective Thermal Properties

Resistance to contact heat was tested according to the standard ISO 12127-1:2016 [22], using the OTI device (OTI Greentech AG, Berlin, Germany) for testing the thermal insulation, at the contact temperatures of 100 and 250 °C. The test principle relies on subjecting the test sample, which is placed on the calorimeter, to the contact with a heating cylinder, heated up to the temperature from the interval 100 to 500 °C. The contact temperature is selected depending on the expected application of gloves at a particular workplace.

In order to carry out the test, three samples should be prepared, with a diameter of 80 mm, and 24 h before the measurement they should be acclimated in the following conditions: the temperature of 20 ± 2 °C and the relative humidity of 65 ± 5 %. During the test, the threshold period time *t*_t_ is measured, which is the time between the first contact with the heating cylinder and the moment when the temperature of the calorimeter increases by 10 °C compared to its initial value. In the case of gloves, the tests are carried out for three samples, and the arithmetic mean is calculated from the three obtained values of the threshold period time *t*_t_. Based on the test results, the gloves are classified to the appropriate efficiency level. The standard EN 407:2004 [23] defines the resistance ranges to contact heat and four efficiency levels, to which protective gloves are classified based on laboratory tests. Each of the effectiveness levels is assigned by a specific contact temperature, *T*_C_. The first level of efficiency corresponds to the resistance to a contact temperature of 100 °C, the second level is 250 °C, and the third is 350 °C, while the fourth level of effectiveness provides resistance to the contact temperature of 500 °C. For each level of effectiveness, the threshold time *t*_t_ should be greater or equal to 15 s.

Radiant heat resistance was determined in accordance with the test method described in ISO 6942:2002 standard (method B) [24], taking into account the guidelines from the EN 407:2004 standard [23]. The principle relies on subjecting the sample of the tested material, which is placed on a suitable holder, to thermal radiation with a flux density of 20 kW/m^2^ at a given time. In the case of protective gloves, the increase of calorimeter temperature by 24 °C is recorded, and at this moment, time is measured. It is expressed in the form of the heat transfer coefficient *t*_24_ in seconds. The tests are performed for two samples, and as a result of the test for gloves, the arithmetic mean was calculated. This is the so-called RHTI24 (Relative Heat Transfer Index), based on which the gloves are classified to one of four levels of effectiveness, according to the standard EN 407:2004 [23]. The first level of protection efficiency is obtained when the coefficient of heat transfer *t*_24_ is greater or equal to 7 s. The second is obtained when the coefficient is greater or equal to 20 s, the third is obtained for the coefficient greater or equal to 50 s, and the fourth is obtained for the coefficient greater or equal to 95 s. A radiant heat resistance test for selected fabric variants was done using a copper calorimeter for heat transfer measurements.

### 2.5. Comfort Thermal Properties

In the case of protective properties, we examine the heat flow from the environment to the body, while in the case of thermal insulation, the properties are examined in the opposite direction: from the body to the outside. The thermal insulation properties of textiles are the most important features of fabrics. They primarily determine the elementary functions of clothing. Besides, thermal insulation properties are important factors in assessing the comfort of clothing for users. The Alambeta testing device (Sensora, Liberec, Czech Republic) was used to determine the thermal insulation properties of the basic and modified basalt fabrics [25,26] when they are the clothing components. The performed test relies on measuring the amount of heat penetrating the tested sample—the fabric, which is placed between the upper plate with a temperature of 35 °C (it should more or less correspond to the human skin temperature) and the lower plate reaching the ambient temperature. During the measurement, metal plates adhere to the tested sample with a pressure of approximately 200 Pa. The device is used in scientific research, and its important advantage is the short time of measurement.

### 2.6. Electrical Properties

As was already mentioned, the material intended for protective gloves (in particular to protect the foundry worker hands) should be classified in the group of anti-static materials. The surface resistances of unmodified and modified basalt fabrics were compared based on the standard ASTM D257-14:2014 [27]. The test method covers direct-current (DC) procedures for the measurement of DC insulation resistance, volume resistance, and surface resistance. From the measurements and geometric dimensions of the material sample and electrodes, the surface resistivity of the material can be calculated. The Keithley Electrometer/High Resistance Meter (Beaverton, OR, USA), Model 6517A, 5½ digit resolution, was used for this purpose. The model can do surface resistivity measurements from 10^3^ to 10^17^ Ω. The resistance was measured by applying a voltage potential across the surface of the insulator sample and measuring the resultant current using the Test Fixture Model 8009 equipped with three concentric electrodes.

### 2.7. Surface Color Assessment

A non-contact digital color imaging system DigiEye (VeriVide, Leicester, UK) was used to carry out the surface analysis of samples. DigiEye provides colorimetric measurements from samples with an ultra-small area, featuring irregular or curved surfaces [19,20,21]. The CIE Standard [28] defined procedures for calculating the coordinates of the CIELAB color space. The Euclidean color difference values based on these coordinates were used for the assessment of basalt fabric before and after the surface modification with chosen coatings.

The total color difference Δ*E* calculated between two points represented by coordinates (*L*_1_, *C*_1_, *h*_1_) and (*L*_2_, *C*_2_, *h*_2_) of specimen is expressed as follows:

(1) $$\Delta E=\sqrt{{\left(\Delta L*\right)}^{2}+{\left(\Delta C*\right)}^{2}+{\left(\Delta H\right)}^{2}}$$

where

(2) $$\Delta C*=C_{1}-C_{2},$$

(3) $$\Delta L*=L_{1}-L_{2},$$

(4) $$\Delta H=2\sqrt{C_{1}-C_{2}}\sin\left(h_{1}-h_{2}\right),$$

wherein, according to [29], *C* is the chroma defined as a colorfulness of an area judged as a proportion of the brightness of a similarly illuminated area that appears as white or highly transmitting; *L* is the lightness defined as the brightness of area judged relative to the brightness of a similarly illuminated area that appears to be white or highly transmitting; *h* is the hue defined as an attribute of visual sensation, according to which the area appears to be similar to one of the perceived colors: red, yellow, green, and blue, or to a combination of two of them.

In general, and independent of the type of color deviation formula, two colors can be optically distinguished if Δ*E* ≥ 1. The total color difference Δ*E* > 3 is perceived as a significant color deviation [30,31]. The CIE illuminant D65 is the most commonly used. The illuminant D65 is a statistical representation of average daylight with a correlated color temperature of approximately 6500 K.

## 3. Results and Discussion

### 3.1. Chemical Composition and Thickness of the Coatings

The scanning electron microscope took images of deposited metal and ceramic coatings on the surface of basalt fabric. For the same samples, the spectroscopy of X-ray energy dispersion was carried out as well. Quantitative and color analyses of the content of individual elements on the basalt fabric surface covered with the 20 μm thick chrome coating and the 18 μm zirconium(IV) oxide coating were carried out. The 20 μm chrome coating deposited on the basalt fabric surface showed 98.92% chrome (Cr) content and 0.62% silicon (Si) content. The 18 μm zirconium(IV) oxide coating deposited on the basalt fabric surface showed 68.24% oxygen (O), 25.68% zirconium (Zr), and 3.25% silicon (Si). It may be related to the analysis of a specific piece of fabric.

### 3.2. Thermal Properties of Fabrics

**Figure 1:**
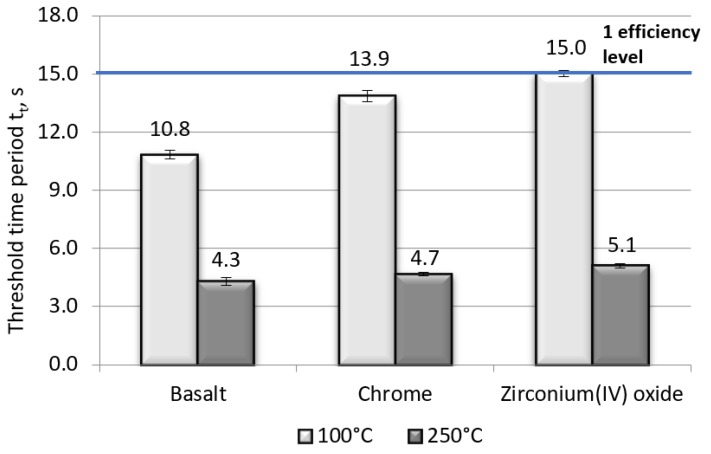
Comparison of contact heat resistance for all the tested materials at contact temperatures of 100 and 250 °C.

The results of contact heat resistance tests and radiant heat resistance were analyzed in terms of the requirements for materials used in protective gloves. The results of contact heat resistance measurements for the contact temperatures of 100 and 250 °C for the fabric made of basalt fiber and its modifications with the metal and ceramics are shown in Figure 1.

The tests of resistance to contact heat at the contact temperatures of 100 and 250 °C showed a significant difference between the values of contact heat for the basic basalt fabric and its modifications. As shown in Figure 1, the value of the tested parameter for the fabric made of basalt fibers for the contact temperature of 100 °C is on the level of 10.8 s, while for the contact temperature of 250 °C, it is equal to 4.3 s. The highest resistance to contact heat for both contact temperatures was obtained by the fabric made from basalt fibers modified with zirconium(IV) oxide coatings. For this sample, at the contact temperature of 100 °C, one level of protection efficiency was achieved, while at the contact temperature of 250 °C, it reached the resistance of 5.1 s to contact heat. For the resistance to contact heat for the contact temperature of 100 °C, the result of 15.0 s belongs to the 95% confidence interval of the average value. None of the samples tested reached the first efficiency level for the resistance to contact heat at the contact temperature of 250 °C.

**Figure 2:**
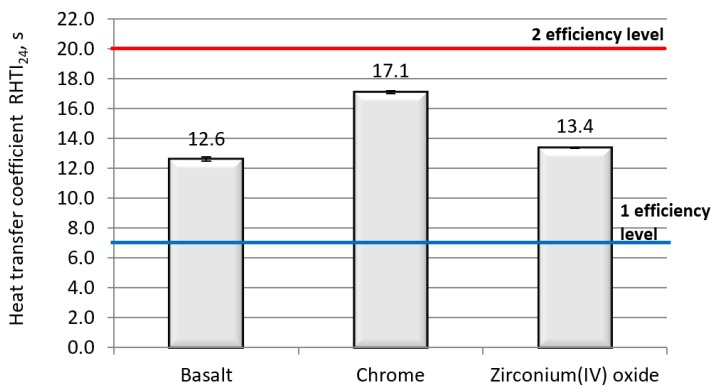
Comparison of radiant heat resistance for all tested materials.

Figure 2 presents the results of thermal radiant resistance measurements for the fabric made of basalt fiber yarns and their modifications.

Based on the test results presented in Figure 2, it is visible that all the tested fabrics have the first level of efficiency of protection against radiant with the 20 kW/m^2^ flux density. The highest resistance of the tested parameter was achieved for the modified basalt fabric with chrome. This is due to the silver, shiny color of the sample. None of the tested samples reached the second level of efficiency of protection against thermal radiation, which in our opinion is not satisfactory.

### 3.3. Comfort Properties of Fabrics

Fabric intended for the production of protective gloves should ensure user comfort. Gloves should protect against high temperatures outside, while maintaining a comfortable temperature inside. Measurements of thermal insulation properties on the Alambeta were performed for the basic (unmodified) fabric made from basalt fibers and for the basalt fabrics modified with chrome and zirconium(IV) oxide coatings. The tested samples of basalt fabric were coated by metal and ceramic only on one side. Since the modified fabrics are not the same from both sides, the right side with coating and left side without coating were tested. The test was carried out under normal climate conditions. Ten measurements were taken for each side of the fabric variant.

**Figure 3:**
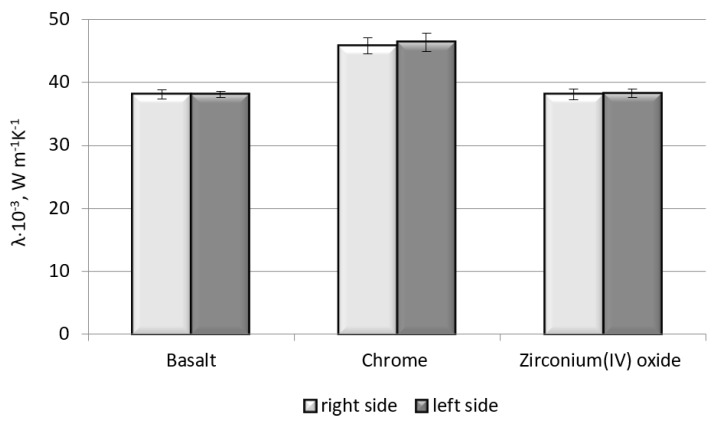
Influence of type of material on the thermal conductivity.

Thermal conductivity determines the material′s ability to conduct heat. Under the same conditions, more heat flows through the material, which is characterized by the higher coefficient of thermal conductivity *λ*. The thermal conductivity of the given material depends on its structure and porosity. Basalt fabric modified with zirconium(IV) oxide obtained approximately the same values of thermal conductivity as the unmodified basalt fabric (Figure 3).

As shown in Figure 3, the highest value of the tested parameter was obtained for the basalt fabric coated with chrome. The results obtained are related to the thermal conductivity coefficient of selected coatings; for the chrome, it is 93.7 W/(m⋅K), while for the zirconium(IV) oxide, it is 2.0 W/(m⋅K).

**Figure 4:**
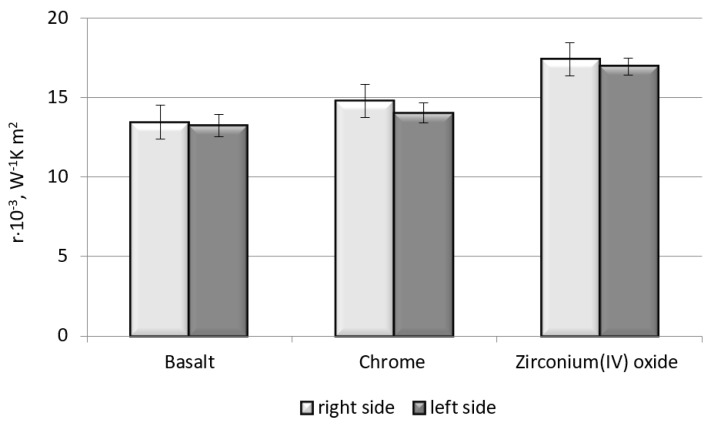
Influence of type of material on the thermal resistance.

Thermal resistance is the ability of a material to resist the flow of heat. The received results are presented in Figure 4.

As shown in Figure 4, the basalt fabric modified with the zirconium(IV) oxide coating showed the highest value of parameter *r*; thus, it provides the best barrier against heat penetrating through the material during the test. Besides, with the higher values of thermal resistance, there is observed an increase of the warmth retention of the tested material and its thickness. A value that is slightly lower than that for the modified fabric with zirconium(IV) oxide of the tested parameter concerning the unmodified basalt fabric was obtained for the chrome-modified basalt fabric.

### 3.4. Electrical Properties of Fabrics

Materials intended for protective gloves are insulation materials. Zirconium(IV) oxide is a dielectric material, but chrome shows a good electrical conductivity. Therefore, the electroconductive properties of coatings applied to the sample surface require assessment. Surface resistance was determined for modified and unmodified basalt fabrics. Surface resistance was equal to 1.9 × 10^12^ Ω/sq for basalt fabric, 1.0 × 10^7^ Ω/sq for basalt fabric modified with a chrome coating, and 3.0 × 10^12^ Ω/sq for basalt fabric modified with a zirconium(IV) oxide coating. Measurements were repeated five times, and mean values were calculated.

The surface resistance values of basalt fabric and basalt fabric modified with the zirconium(IV) oxide are comparable. The results place the products in the group of anti-static materials. An increase in the electrical conductivity was noticed in the case of the chrome coating basalt fabric compared to the unmodified basalt fabric. Therefore, it belongs to the group of static dissipative materials and is classified as an anti-static material. Such materials can protect the hands of the user and can prevent electrostatic discharge.

### 3.5. Surface Color Assessment of Fabrics

**Figure 5:**
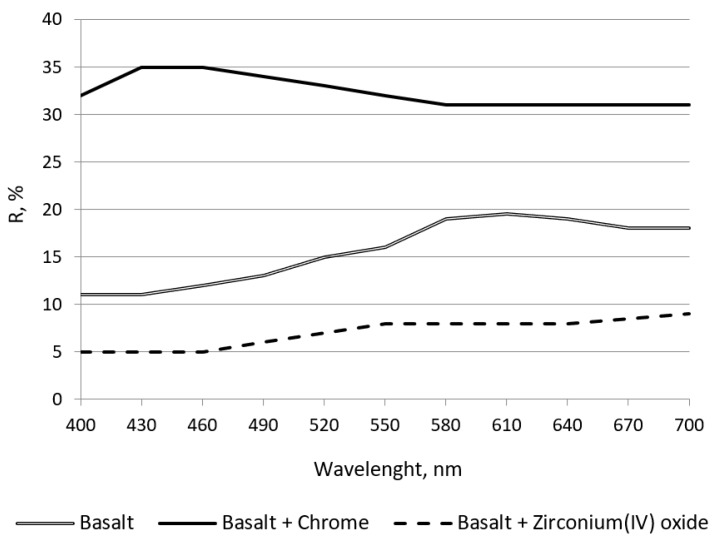
Remission curves of unmodified and modified basalt fabrics.

Colorimetric measurements of the sample surfaces were made to evaluate the quality of the fabric surface coating. The wavelength region of interest encompassed the electromagnetic energy of wavelengths from approximately 400 nm (violet) to 700 nm (red). Remission curves obtained for the unmodified basalt fabric and basalt fabric modified with the chrome coating and basalt fabric modified with the zirconium(IV) oxide are shown in Figure 5.

The results of the remission measurement of samples in the spectrum of 400–700 nm indicate a significant effect of applied coatings on the remission measurement. The smallest value of spectral remission factor *R* is noticed for the basalt fabric coated with zirconium(IV) oxide. The highest value of factor *R* is noticed for the basalt fabric coated with the chrome. Therefore, the sample works better in the hot work environment due to a larger stream of reflected light from the tested surface.

**Figure 6:**
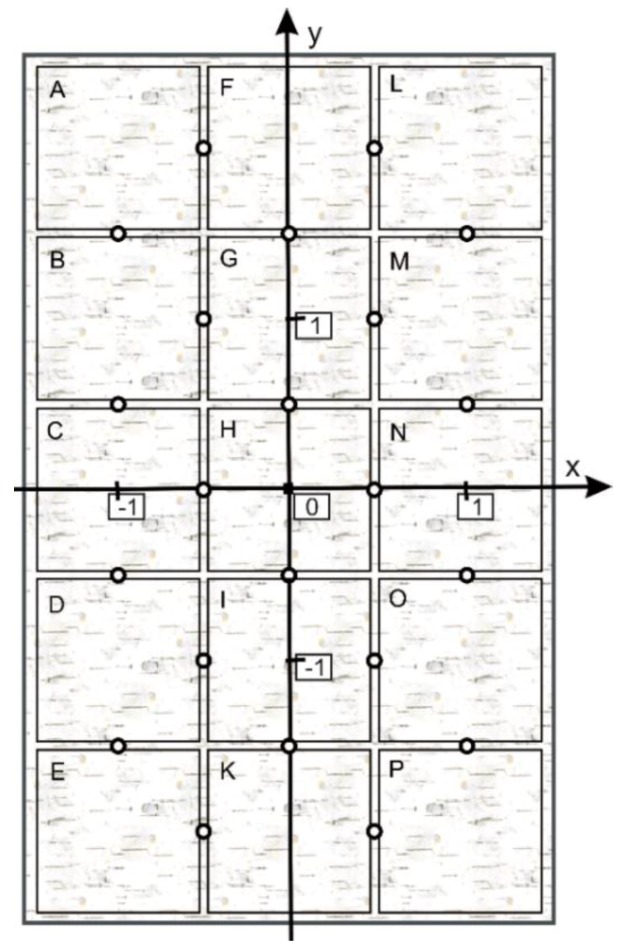
Squares arrangement on the sample surface.

The following assumptions have been made. (a) The sample lies on an XY plane in such a way that its center corresponds to the coordinates (0,0); (b) the assumed measuring surface of the sample was divided into 15 squares with length sides of 1 cm (Figure 6). Each square covers part of the unmodified or modified basalt fabric.

Coordinates (*x*,*y*) were assigned to each pair of squares according to Table 3.

**Figure 7:**
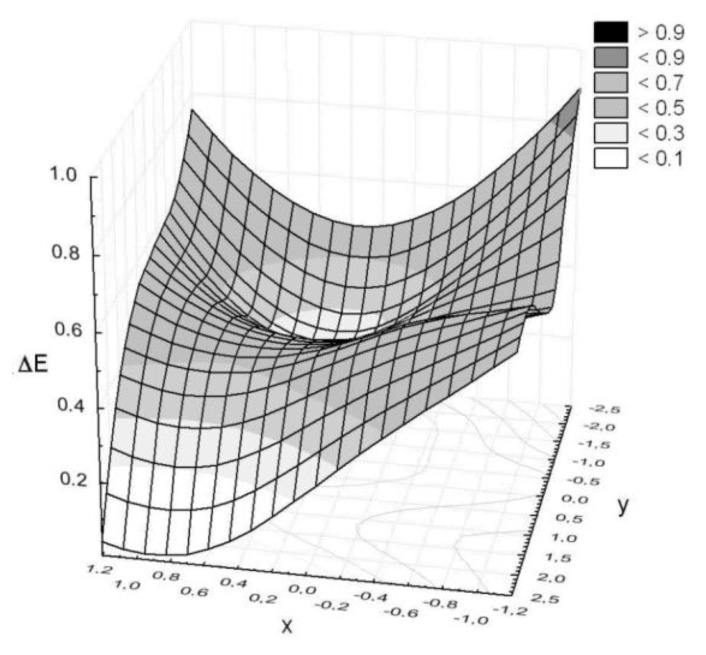
Color differences observed on the basalt fabric surface.

**Figure 8:**
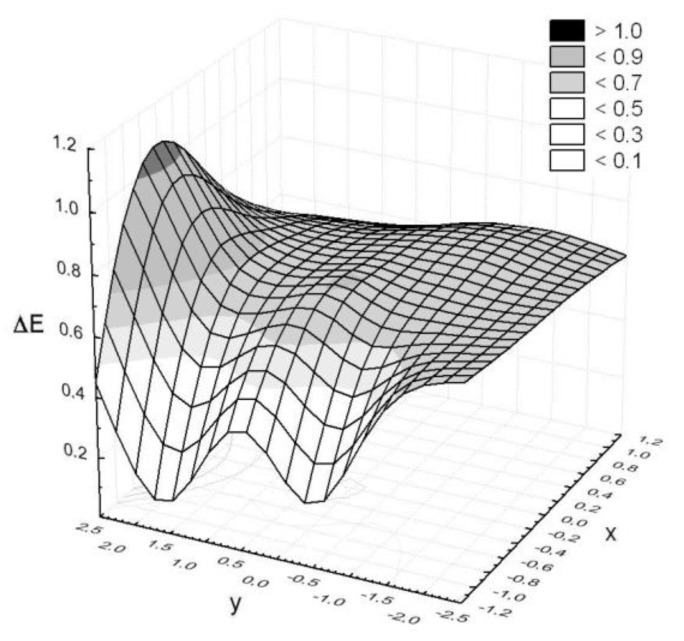
Color differences observed on the basalt fabric surface modified with the chrome coating.

**Figure 9:**
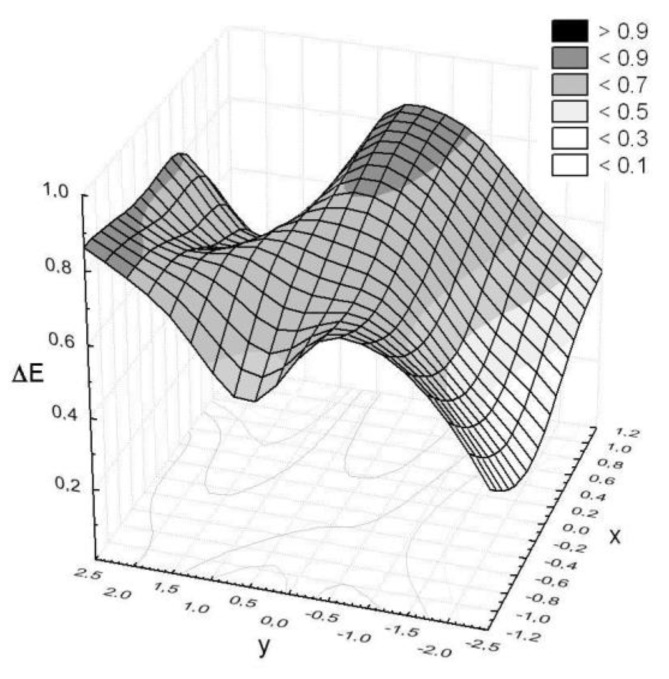
Color differences observed on the basalt fabric surface modified with a zirconium(IV) oxide coating.

The colors on DigiEye of two neighboring squares were compared based on the instrumental color measurement. Differences Δ*E* that were determined for selected pairs of squares were aimed at assessing the impact of cover on the chosen properties of the basalt fabric. A controlled illumination cabinet with D65 illuminant was chosen. The measurement results are presented in Figure 7, Figure 8 and Figure 9 in a form of surface 3D plots. The distance-weighted least squares method was used to fit a curve to the data. The received results presented in Figure 7, Figure 8 and Figure 9 indicate that the sample surfaces are uneven. The unevenness was assessed based on the variation coefficient of the total color difference.

The obtained results show the variation coefficient below 30% (Table 4). It means that the tested surfaces are uneven, but the values of Δ*E_av_*, and additionally Δ*E_max_* below 1, indicate that the pairs of colors recognized on the sample surface are not optically distinguished. Moreover, the unmodified basalt fabric is characterized by a 24% variation coefficient of the total color difference.

Unmodified and modified samples were also compared. The total color differences Δ*E* for pairs of samples were determined. A Δ*E* value equaling 11.8 was received for the pair of basalt fabric and basalt fabric modified with the zirconium(IV) oxide coating. The lower value of Δ*E* equal to 7.7 was received for the pair of basalt fabric and basalt fabric modified with the chrome coating. The value of Δ*E* above 3 is perceived as a significant color deviation.

## 4. Conclusions

The modification of basalt fabric surface with the metal and ceramics using the magnetron sputtering technique in the field of thermal properties tests affected the results of tests carried out. Better thermal properties were obtained for modified basalt fabrics compared to unmodified fabric. The first level of protection against contact heat for the contact temperature of 100 °C was obtained for the zirconium(IV) oxide modified basalt fabric. None of the modified fabric samples reached the second level of effectiveness against contact heat at the contact temperature of 250 °C. The highest value for the heat radiant resistance test was obtained for the chrome-modified basalt fabric. However, no tested sample reached the second level of protection against heat radiation. Modified and unmodified basalt fabrics belong to anti-static materials. Based on the results of colorimetric measurements, for the chrome-modified sample, it was noticed that a larger stream of light is reflected from the tested surface. The lowest value of thermal conductivity, and thus the higher value of thermal resistance, was obtained for the basalt fabric coated with the zirconium(IV) oxide.

The results obtained using the digital color imaging system DigiEye indicated that the samples’ surface was uneven. The level of variation in the coefficient value of the total color difference was below 30%.

The following conclusions can be drawn from the research:

- The metal and ceramics can be applied as a coating on the surface of basalt fabric.
- The modification of basalt fabric with the zirconium(IV) oxide and chrome coatings enables the improvement of its thermal properties.
- In order to reach a compromise between providing adequate protective and functional properties of material intended for protective gloves, further research will be carried out.

## Figures and Tables

**Table 1:** Images of tested fabrics.

Fabric Description	Fabric Made of Basalt Fibers	Basalt Fabric Modified with a Chrome Coating 20 µm	Basalt Fabric Modified with an Zirconium(IV) Oxide Coating 18 µm
Fabric image	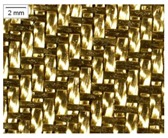	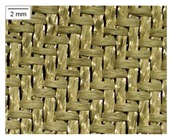	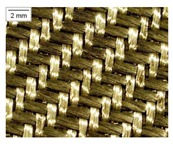

**Table 2:** Deposition parameters of chrome or zirconium(IV) oxide coatings manufactured with the use of magnetron sputtering.

Coating	Target Material	Magnetron Sputtering Power/kW	Argon Pressure/Pa	Oxygen Partial Pressure/Pa	Deposition Time/ks
Chrome	Chromium (4N)	3	0.5	-	26.4
Zirconium(IV) oxide	Zirconium (2N5)	3	0.5	0.04	37.6

**Table 3:** Coordinates corresponding to a pair of squares.

**Pair of Squares**	**AF**	**FL**	**BG**	**GM**	**CH**	**HN**	**DI**	**IO**	**EK**	**KP**	**AB**
*x*	−0.5	0.5	−0.5	0.5	−0.5	0.5	−0.5	0.5	−0.5	0.5	−1.0
*y*	2.0	2.0	1.0	1.0	0.0	0.0	−1.0	−1.0	−2.0	−2.0	1.5
**Pair of Squares**	**BC**	**CD**	**DE**	**FG**	**GH**	**HI**	**IK**	**LM**	**MN**	**NO**	**OP**
*x*	−1.0	−1.0	−1.0	0.0	0.0	0.0	0.0	1.0	1.0	1.0	1.0
*y*	0.5	−0.5	−1.5	1.5	0.5	−0.5	−1.5	1.5	0.5	−0.5	−1.5

**Table 4:** Results of samples surface analysis.

Parameters	Basalt Fabric Modified with a Chrome Coating	Basalt Fabric	Basalt Fabric Modified with an Zirconium(IV) Oxide Coating
Δ*E_max_*	0.9	0.6	0.9
Δ*E_av_*	0.6	0.4	0.6
Variation coefficient	24%	29%	28%
